# A cross-sectional study on the knowledge of and interest in Planetary Health in health-related study programmes in Germany

**DOI:** 10.3389/fpubh.2022.937854

**Published:** 2022-10-31

**Authors:** Vera Klünder, Paula Schwenke, Elke Hertig, Carmen Jochem, Irena Kaspar-Ott, Eva-Maria Schwienhorst-Stich, Lisa Stauch, Michaela Coenen

**Affiliations:** ^1^Institute for Medical Information Processing, Biometry, and Epidemiology–IBE, Chair of Public Health and Health Services Research, LMU Munich, Munich, Germany; ^2^Pettenkofer School of Public Health, Munich, Germany; ^3^Faculty of Medicine, Regional Climate Change and Health, University of Augsburg, Augsburg, Germany; ^4^Department of Epidemiology and Preventive Medicine, Faculty of Medicine, University of Regensburg, Regensburg, Germany; ^5^Department of General Practice/Family Medicine, University Hospital Würzburg, Würzburg, Germany; ^6^Teaching Clinic of the Faculty of Medicine and Institute of Medical Teaching and Medical Education Research, University of Würzburg, Würzburg, Germany

**Keywords:** climate change, Planetary Health, student, health, higher education

## Abstract

Planetary Health connects human health with the natural and anthropogenic systems on which it depends. Planetary Health education has been growing in a wide range of health-related disciplines, yet not been widely implemented in health-related university curricula. This cross-sectional study focused on students' knowledge of and interest in Planetary Health education in order to assess the relevance of Planetary Health and Planetary Health topics for university students and their fields of study. We surveyed 1,303 students enrolled in health-related programmes in Bavaria, Germany. Data was collected on students' previous knowledge of and interest in Planetary Health, as well as the relevance of different Planetary Health topics and students' willingness to select a Planetary Health elective within their study programmes. Descriptive statistics were calculated. The majority of participants (73.8%) had not yet heard of Planetary Health but were interested in learning more about this field (90.7%). Most participants considered Planetary Health as relevant to their field (81.6%) and would likely choose a Planetary Health elective (81.9%). Participants were most interested in topics about general associations between climate and health as well as its connections with mental health and (micro) plastics. There is an urgent need and high student interest to implement a Planetary Health module in health-related study programmes in order to move this topic more into focus for the next generation of students.

## Introduction

Planetary Health describes a concept that relates human health with the natural systems on which it depends in relation to political, economic and social systems. While human health has been improving over the last centuries, especially with regards to increased life expectancy and lower infant mortality due to progress in health care, this change has led to the exacerbation of our planet‘s natural systems (1). In 2009, Rockstrom and colleagues defined nine planetary boundaries (e.g., climate change and freshwater use) as thresholds within which humans and human health can flourish for current and future generations if they are not crossed (2). While the strains on these boundaries are growing, their negative effects on human health are also increasing. For instance, heat waves are associated with increased mortality risk (3), ocean acidification can lead to health risks associated with seafood consumption (4) and biodiversity loss impacts the spread of infectious diseases (5). These multi-faceted impacts on human health call for a holistic approach which the interdisciplinary field of Planetary Health offers. This systemic perspective involves the “understanding that human health outcomes emerge from complex interactions between natural and social systems and that stakeholder engagement is necessary in the coproduction of this knowledge” (6). Therefore, across a wide range of disciplines, the interest in Planetary Health education is emerging and systems of the future which influence population health, e.g. healthcare systems, city planning, and their employees, must be prepared to deal with evolving health risks (7). In addition, following the principle of “primum non nocere” the health sector and other sectors must minimize their ecological footprint and thus not contribute further to the causes of the problem. As potential communicators between policy, science, practice and society, and as the most trusted members of society, health professionals are key persons in leading transformative changes for Planetary Health (8). The need for transformative changes ranges from the micro level regarding direct patient or client contacts (e.g., doctor-patient relationship) to the macro level regarding policies of the health sector, as well as the six building blocks of health systems (9), among others.

Educational institutions such as universities have a major responsibility to embed Planetary Health education in their curricula and lay the groundwork for urgently needed transformative changes. Students and employees in the health system should be trained to become change agents who actively drive the achievement of sustainable health goals (10). These changes are already in motion internationally as calls for the implementation of Planetary Health into medical curricular increase (11, 12) and countries such as the UK show (13) how Planetary Health has been successfully integrated into curricular. Additionally, initiatives such as the “Planetary Health Report Card Initiative” show students' engagement in sharing implementation efforts of Planetary Health into their education worldwide (14). Furthermore, guidelines on implementing Planetary Health education have been published, such as the AMEE Consensus Statement, in order to support the implementation of Planetary Health into curricular (15). To our knowledge, in Germany there are still a limited number of educational institutions addressing Planetary Health.

In order to promote Planetary Health education in Germany, we conducted a survey to gauge students' familiarity with basic Planetary Health concepts as well as their interest in the integration of these concepts in the form of an elective course module, as we were in the process of developing an elective course module which will be available to a wide range of students in Bavaria, Germany.

## Methods

### Study design

This cross-sectional study was designed as an online survey among university students enrolled in (health-related) study programmes in the federal state of Bavaria, Germany, located in the southeast with a population of about 13 million people and is thus one of the largest federal states in Germany. We conducted this study in Bavaria as we will use the study results for the conceptualization and development of a Planetary Health elective to be implemented at Bavarian universities. The study protocol was approved by the Ethical Committee of the Medical Faculty of LMU Munich (Project Nr: 21-0973 KB, September 28, 2021).

### Sample and recruitment strategy

The following inclusion criteria were applied in this study: participants should be (1) aged 18 years and above and (2) enrolled in a (self-defined) health-related field of study at a Bavarian university. The study population was derived using a sampling strategy combining voluntary and snowball sampling. We carried out a thorough search on health-related study programmes at Bavarian universities using the websites “Hochschulkompass.de” and “Studieren-in-Bayern.de”. The identified study programmes cover a wide range of health-related programmes such as medicine, nursing science, psychology, health pedagogics, public health and dentistry. The list of these study programmes along with the universities offering these programmes is shown in the Supplementary Table 1. A contact person or student representative was identified for each study programme, in most cases degree coordinators or faculty bodies, to whom an invitation email including the link to the survey was sent in the first week of October 2021. Contact persons and student representatives were encouraged to also spread the survey invitation to other fields for whom they saw Planetary Health as relevant. A reminder was sent three weeks later. Additionally, the link was shared on social media via accounts of health-related study programmes to reach out to students of other fields of study who also saw Planetary Health as relevant to their studies. Participation was therefore not promoted for other fields but participation was not limited to predefined health-related fields of study and completion of the survey was possible for students of other fields. This method was used in order to allow a broader spectrum of students to participate and enable us to find further fields of study that envisage Planetary Health education to be relevant to their curriculum.

### Material and data collection

The survey was developed by a group of researchers in health-related fields from three Bavarian universities with expertise in Planetary Health education (LMU Munich, University of Augsburg, University of Regensburg). The rationale for the development of this survey was to inform the conceptualization and content of a Planetary Health elective for a broad range of health-related study programmes. Closed and open-ended questions were included to assess previous knowledge of and interest in Planetary Health as a discipline, as well as first experiences with and importance of Planetary Health education from the students' perspective. Those with previous knowledge of Planetary Health were asked to define Planetary Health in their own words. We also included a question on students' willingness to choose a Planetary Health online elective in the future. Questions about students' previous knowledge of and interest in Planetary Health were designed to give an overview of the status quo and potential interest in a Planetary Health elective. The questions regarding the interest in its topics were based on Planetary Health course syllabuses identified in the literature and further research on additional topics.

The survey with its questions was discussed and iteratively refined over a period of three weeks by the team of ten researchers, including students enrolled in health-related studies and was tested and piloted among the same team. It was conducted in German; thus, questions and answers were translated to English for this paper. The study questionnaire is shown in the Supplementary Table 2.

Data collection was carried out using Lime Survey. The survey started on October 1, 2021 and ended five weeks later on November 8, 2021. A total of 1,479 students participated in the survey of whom 1,303 (88%) filled in all questions of the survey and were included in data analyses.

### Statistical analyses

Statistical analyses were done in SPSS 27 (16). Descriptive statistics were calculated for closed questions. To allow for better comparison between different fields of study, study programmes were grouped into overarching fields of study for most of the analyses (e.g., Midwifery, Nursing Science, Speech Therapy and Physiotherapy were allotted to Applied Health Sciences). The list of study programmes along with their fields of study is shown in Table 1.

**Table 1:** Participant characteristics (*N* = 1,303).

		**N (%)**
Gender		
	Male	276 (21.2%)
	Female	1,001 (76.8%)
	Diverse	6 (0.5%)
	No answer	20 (1.5%)
Age group		
	18–22 years	602 (46.2%)
	23–27 years	445 (34.5%)
	28–32 years	146 (11.2%)
	33–37 years	45 (3.5%)
	38–42 years	21 (1.6%)
	43 years and above	36 (2.8%)
	No answer	8 (0.6%)
Study programme		
	Applied Health Sciences	125 (9.6%)
		Speech Therapy 5 (0.4%) Midwifery 19 (1.5%) Nursing Science 49 (3.8%) Physiotherapy 52 (4.0%)
	Dentistry	73 (5.6%)
	Epidemiology/Public Health	95 (7.3%)
		Epidemiology 5 (0.4%) Public Health 90 (7.0%)
	Health Sciences	71 (5.4%)
		(Applied) Healthcare Research 2 (0.2%) Healthcare Management 12 (1.0%) Health Sciences 54 (4.1%) Medical Research 3 (0.2%)
	Medicine	629 (48.3%)
	Natural Sciences	40 (3.1%)
		Biology 3 (0.2%) (Biomedical) Neuroscience 3 (0.2%) Chemistry 3 (0.2%) Engineering 1 (0.08%) Geography 1 (0.08%) Global Sustainability Science 1 (0.08%) Mechanical Engineering 1 (0.08%) Molecular Medicine 25 (2.0%)
		Physics 1 (0.08%) Water and Environmental Engineering 1 (0.08%)
	Pharmacy	50 (3.8%)
	Psychology	105 (8.1%)
	Social Sciences/Humanities	71 (5.4%)
		Communication Science 4 (0.3%) Cultural Studies 1 (0.08%) Deaconry 2 (0.2%) Theology 1 (0.08%) Pedagogy/Educational Science 16 (1.2%) Philosophy 3 (0.2%) Political Sciences 6 (0.5%) Social Economics 7 (0.5%) Social Management 1 (0.08%) Social Work 8 (0.6%) Sociology 5 (0.4%) Teaching 17 (1.3%)
	Veterinary Medicine	32 (2.5%)
	Other	12 (1.1%)
		Business Law 1 (0.08%) Business Studies 1 (0.08%) Other 10 (0.8%)
Degree level of programme enrolled in		
	Bachelor	296 (22.7%)
	Master	172 (13.2%)
	PhD	16 (1.2%)
	State examination	788 (60.5%)
	Other/No answer	31 (2.4%)

Contingency tables were calculated for “previous knowledge of Planetary Health” and “age group” as well as “previous knowledge of Planetary Health” and “study programme”, respectively. For the participants who had previously heard of Planetary Health, contingency tables were calculated for their fields of study and previous contact with Planetary Health topic in their study programmes.

Data from free text questions and open-ended questions were analyzed by grouping them into overarching themes. The first author coded the answers to open-ended questions and grouped codes to overarching themes. A second author (PS) cross-checked the codes and overarching themes.

## Results

In total, we received 1,303 fully completed questionnaires. As shown in Table 1, the majority of participants were female (*n* = 1,001; 76.8%), aged between 18 and 27 years (*n* = 1,047; 80.7%) and enrolled in the field of medicine (*n* = 629; 48.3%). The gender proportion of medical students in Germany in 2021 (67.1%) (17) is similar with the one in our sample (76.8%). Due to our sampling strategy a sample size calculation and calculation of the response rate was not possible.

Almost three quarters (*n* = 962; 73.8%) of the participants had not yet heard of Planetary Health as a discipline, but 90.7% (*n* = 1,182) wanted to learn more about this topic. In total, 81.6% of the participants (*n* = 1,064) considered the implementation of Planetary Health topics in their studies as very important (*n* = 433; 33.2%) and rather important (*n* = 631; 48.4%), respectively. In total, 81.9% of participants (*n* = 1,067) reported that they would likely choose a Planetary Health online elective if it were available at their university (see Table 2).

**Table 2:** Previous knowledge of and interest in Planetary Health, as well as importance of and willingness to choose a Planetary Health online elective (*N* = 1,303).

			***N* (%)**
Previous knowledge of Planetary Health			
	Yes		314 (24.1%)
	No		962 (73.8%)
	No answer		27 (2.1%)
Interest in learning more about Planetary Health			
	Yes/Rather yes		1,182 (90.7%)
		Yes	726 (55.7%)
		Rather yes	456 (35.0%)
	Rather No/No		99 (7.6%)
		Rather no	76 (5.8%)
		No	23 (1.8%)
	No answer		22 (1.7%)
Importance of implementing Planetary Health topics into curricula			
	Very important/Rather important		1,064 (81.6%)
		Very important	433 (33.2%)
		Rather important	631 (48.4%)
	Rather unimportant/Unimportant		185 (14.2%)
		Rather unimportant	151 (11.6%)
		Unimportant	34 (2.6%)
	No answer		54 (4.1%)
Willingness to choose a Planetary Health online course, if it were available as an elective			
	Yes/More likely		1,067 (81.9%)
		Yes	456 (35.0%)
		More likely	611 (46.9%)
	Less likely/No		199 (15.3%)
		Less likely	149 (11.4%)
		No	50 (3.8%)
	No answer		37 (2.8%)

Participants who had previously heard of Planetary Health, were asked to define the discipline Planetary Health. Of the 314 (24.1%) students with previous knowledge of Planetary Health, 227 students (72.3%) defined Planetary Health in their own words. We categorized these answers into eight overarching themes and definitions (see Table 3).

**Table 3:** Students' definition of Planetary Health (*N* = 227).

**Definition of Planetary Health**	***N* (%)**
Relation of environment/climate (change), (all) living beings and the planet and human health	102 (44.9%)
Relation between healthy humankind and a healthy planet	47 (20.7%)
Health of the planet	24 (10.6%)
World health (understood as a concept similar to global health)	23 (10.1%)
Climate protection, environmental protection or sustainability for the planet	13 (5.7%)
Life and health within the planetary boundaries	9 (3.9%)
Climate-friendly diet	3 (1.3%)
Others	6 (3.0%)

**Figure 1:**
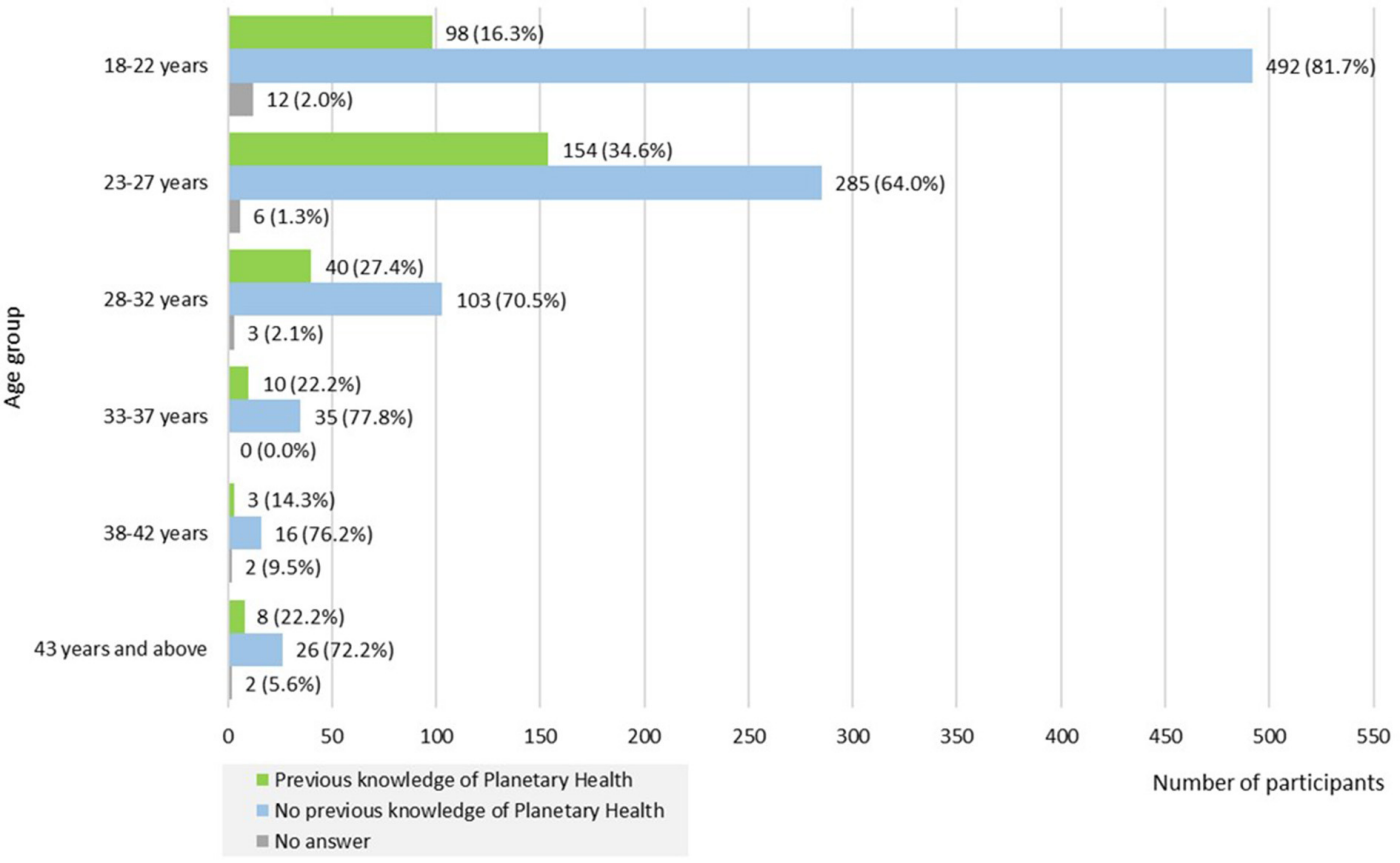
Previous knowledge of Planetary Health stratified by age group (*N* = 1,303).

We found a difference regarding age and previous knowledge of Planetary Health in our sample. More than a third of respondents (*n* = 154; 34.6%) aged 23–27 years had already heard of Planetary Health compared to only 16.3% (*n* = 98) of those aged 18–22 years. No differences were found regarding age and interest in Planetary Health (see Figure 1).

**Figure 2:**
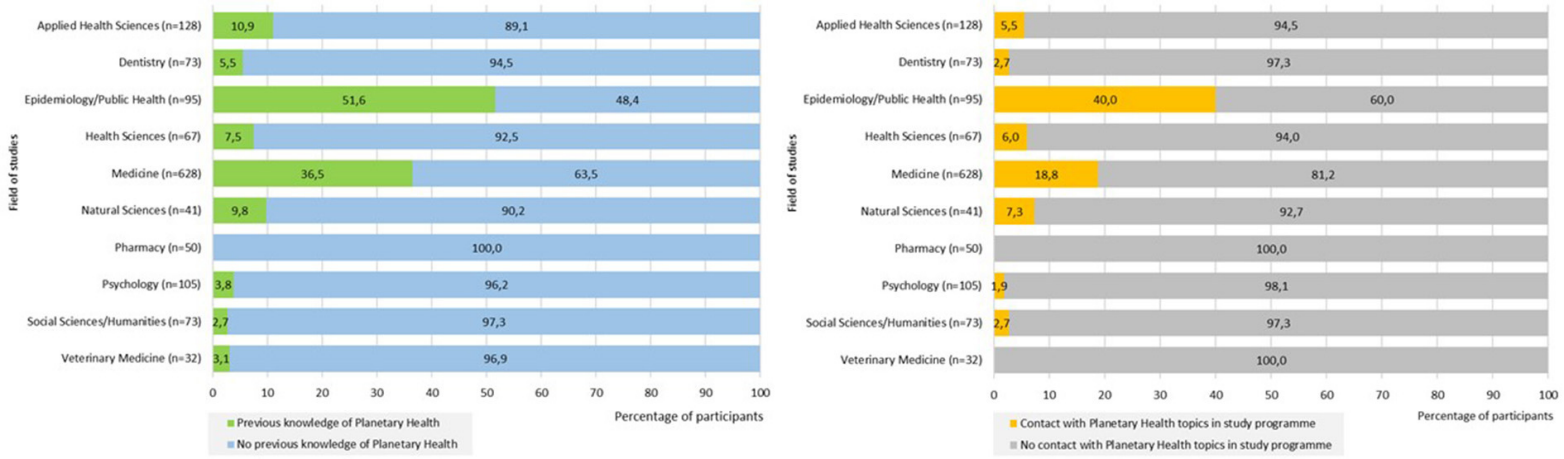
Previous knowledge of and contact with Planetary Health by field of study (*N* = 1,303).

A clear difference was found in previous knowledge of Planetary Health regarding the participants' fields of study. Participants studying medicine, epidemiology and public health, respectively were more likely to have previously heard of Planetary Health compared to other fields of study (see Figure 2). Of the participants which had previously heard of Planetary Health, mainly participants studying medicine (*n* = 118; 18.8%), epidemiology and public health (*n* = 38; 40.0%) and natural sciences (*n* = 4; 9.8%) had previously come into contact with the topic of Planetary Health in the university context.

**Figure 3:**
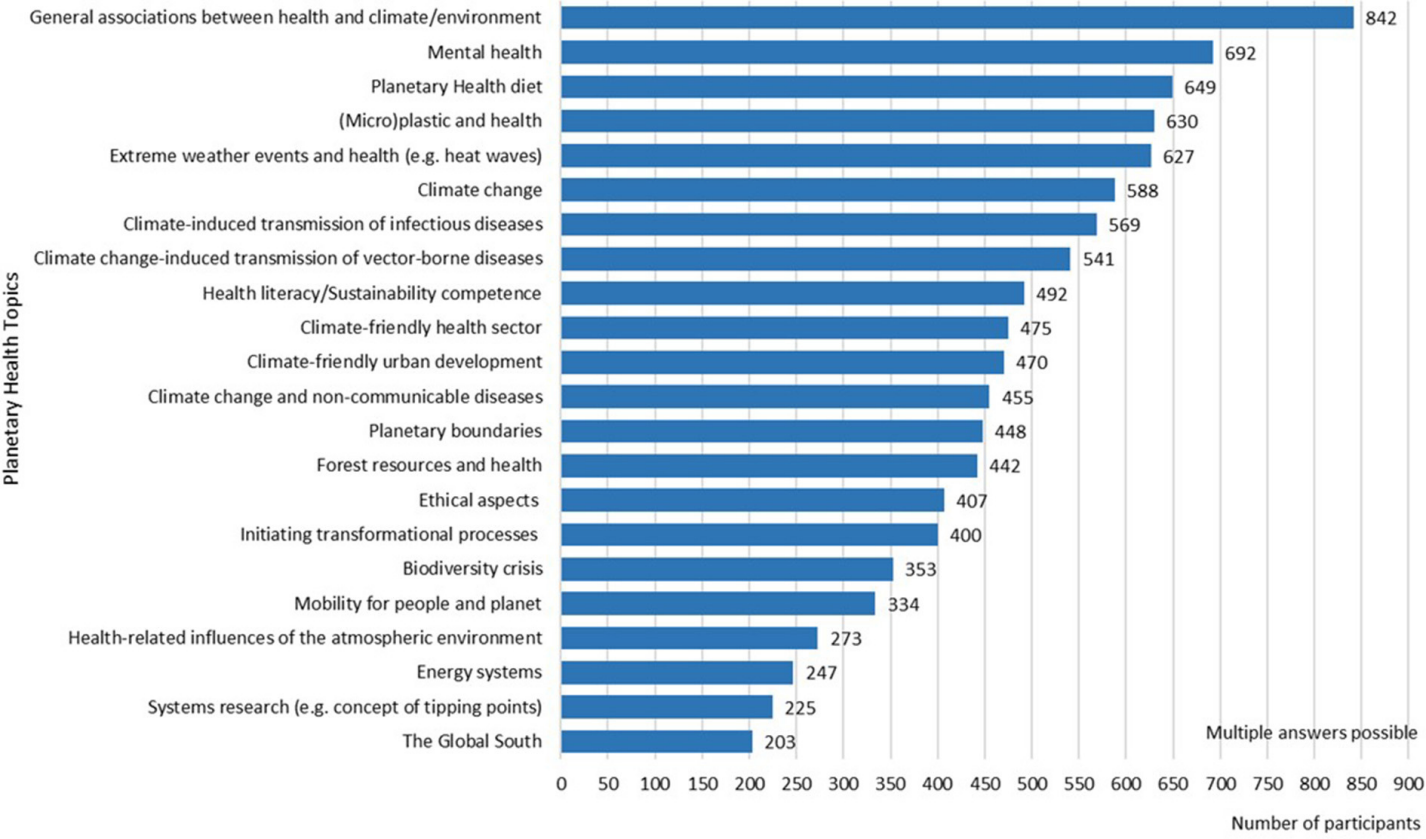
Participants' interest in Planetary Health topics (*N* = 1,303).

Regarding the interest in Planetary Health topics, participants chose up to six of a total of 22 suggested topics for which they had the most interest. The majority of participants showed interest in learning more about the “General Associations between Health and Climate/Environment” (*n* = 824; 65.0%) (see Figure 3). The topics “Mental Health” (*n* = 692; 53.1%), “Planetary Health Diet” (*n* =649; 50.0%), “(Micro) Plastics and Health” (*n* = 630; 48.3%) and “Extreme Weather Events and Health (e.g., heat waves)” (*n* = 627; 48.1%) were also seen as interesting topics by almost half of the participants. At the end of this question, there was also an option to suggest further topics; 124 students named a wide variety of additional topics of interest. The most frequently named topics were the following: “Political and economic dimensions and structures”, “Focus on solutions with approaches and preventive measures”, and “Becoming active as a health care worker”.

Concerning the interest in specific topics, clear differences within health-related fields of study were found. Students of midwifery (*n* = 19) were particularly interested in certain topics, such as Extreme Weather Events, Climate-induced Transmission of Infectious Diseases, (Micro)Plastics and Health, Planetary Health Diet, Mental Health, Forest Resources and Health and a Climate-friendly Health Sector. Students of Natural Sciences (*n* = 40) were strongly interested in Climate Change, Energy Systems, System Research, Biodiversity Crisis and Planetary Boundaries and students of Social Sciences and Humanities (*n* = 71) were mostly interested in Mental Health, Climate-friendly Urban Development, Health-literacy/Sustainability Competences and Ethical Aspects of Planetary Health.

## Discussion

Our survey confirmed a wide-spread interest in the concept of Planetary Health and its potential integration into a variety of health- and science-based specialties. The results suggest that there is wide-spread awareness if not of Planetary Health *per se*, then at least of environmental degradation and threats which have differing relevance depending on the respondents' field of interest. This growing interest is confirmed by top-down directives such as the recent release of guidelines (15). Our results show that the majority of students have no previous knowledge of Planetary Health as a discipline. Furthermore, only about one tenth of the study participants had experience with Planetary Health related topics within their study programmes. These results are in line with previous studies. An international study found that Planetary Health education was incorporated into only 15% of medical students (18). However, the interest in learning more about Planetary Health, as well as its relevance for their fields of study is exceptionally high in the surveyed population indicating an urgently needed adaptation of study programmes. Previous studies had already demonstrated the impact of incorporating Planetary Health into health-related fields of study by framing health within a wider understanding of the interdependent influences of human and Planetary Health and focusing on how future health professionals can, for example, contribute to the decarbonisation of the health sector (19).

In our study, the students' overall level of knowledge and previous involvement in Planetary Health courses in their study programmes was low, but a clear difference was found between students of epidemiology, public health and medicine vs. students of other health-related fields of study, which was much lower. Where Planetary Health education is integrated, it mainly focuses on medical students, doctors, nursing students, and nurses (20). A university in England, for instance, has implemented “evidence-informed sustainability scenarios” into its nursing curriculum leading to increased awareness regarding the nurses' waste management (21).

However, Planetary Health is relevant for a wider field of health-related studies. In line with the São Paulo Declaration on Planetary Health, it is crucial to “incorporate planetary health concepts and values into the main curricula and professional codes of conduct for all future health practitioners” (22). For instance, midwives play an important role during sensitive stages of life, namely before, during and after the birth of a child. They are in regular and very intensive contact with parents during a vulnerable phase of the transition to becoming a family. Counseling parents on Planetary Health issues (for example regarding co-benefits of healthy and sustainable lifestyles for both human health and the state of natural systems) during the window of opportunity that a child's birth offers has a large potential for Planetary Health related transformative processes. The International Confederation of Midwives states that “Midwives can take an essential role in influencing social change in relation to sustainability” (23). Additionally, midwives can play a significant role in addressing the impact of extreme climate events on the access to maternal, newborn and child health care services. They (along with other health professionals) are also trusted messengers who have the potential to raise local awareness and to initiate climate change related and transformative actions and processes. Thus, students of midwifery and practicing midwives should be specifically addressed and included in education targeting for Planetary Health. In line with this, target-group specific learning formats that combine knowledge practices in midwifery with the existing scientific evidence regarding Planetary Health should be further developed and provided.

Our study shows that besides the interest in the general connections between health and climate/environment, the topics of mental health, planetary health diet and (micro)plastics were of exceptionally high interest for students. The large interest in general connections between health and climate/environment is unsurprising and can be explained by the fact that most study participants had not previously heard of Planetary Health and are therefore firstly interested in gaining a general overview on and understanding of the topic Planetary Health, rather than focusing on specific aspects of Planetary Health.

The importance and scientific discussion of mental health related issues have significantly increased in the last decades. Adolescent mental health has also worsened in many countries during the 21st century (24). Climate change is a contributing factor for the decline of mental health and well-being, and future generations will face the challenges of climate change related events affecting their well-being and mental health. Examples of the affect of climate change on mental health can be seen directly through natural disasters, indirectly by consequences of climate change that affects social, economic and environmental aspects of life, and comprehensively through its global consequences. Additionally, physical health problems may impact mental health (25). Awareness of the importance of mental health and interest in addressing this issue have thus also increased in recent years. The fact that interest in mental health is high in our survey may also be amplified by the effect of the current COVID-19 pandemic. Numerous studies have found that the pandemic has had an additional negative impact on mental health, especially on children and adolescents that are at crucial phases of development (26). The combination of generally increased awareness of mental health in recent decades and the pandemic situation in which our survey took place may be why the topic of mental health was given such a prominent place in this survey.

Our results also show a strong interest by students on the topic of (micro)plastics and health. There is growing evidence about the ubiquitous existence of plastic in the environment and in human (and animal) bodies (27). Possible negative health effects like inflammation, cell toxicity, alterations of immune response and endocrine disruption are discussed. However, the cause-effect relationships and direct health effects are still unknown (28, 29). It is a topic present in everyday consumption choices, including the labeling of food and personal hygiene products as free of microplastics. It can be assumed that the combination of increased awareness about this (possible) health hazard of unknown magnitude, the presence in everyday consumption choices as well as the current gaps in scientific evidence about the health hazards of (micro)plastics result in this strong interest among the respondents.

Although this study mainly focused on students from health-related fields, a considerable number of participants came from other fields of study such as social sciences and natural sciences. While the implementation of Planetary Health education is of crucial importance within health-related studies, the interconnected nature of environmental, social and health challenges we face today calls for an integration of Planetary Health topics in other disciplines. Five foundational domains were identified within the Planetary Health education framework: i.e., interconnection with nature, the Anthropocene and health, systems thinking and complexity, equity and justice as well as movement building and systems change (10). Additionally, the need for transdisciplinary approaches in order to tackle the topic of Planetary Health has increasingly been recognized in research and education (30). This supports the idea for the need to transformation of education from traditional disciplinary thinking toward a truly inter- and transdisciplinary education, broadening Planetary Health further than only health-related studies. However, considering the partly strong persistence of traditional disciplines, the integration of Planetary Health education in existing social and natural sciences can also represent a strategy to establish the concept, for instance in the fields of environmental humanities, environmental economics and law, as well as in climate and environmental studies. Geography, a diverse discipline in itself, represents a study par excellence to integrate with Planetary Health education. Care should be taken however that the implementation does not stay within academic faculties or on a weak interdisciplinary level, but that it evolves into true inter- and transdisciplinary integration.

Results from the survey will be used to develop an interdisciplinary online elective designed to empower students in health-related study programmes to move beyond the “old ways” of thinking and existing patterns to new ways of strengthening knowledge and skills which aim to protect and restore the health of these systems. Even though awareness for Planetary Health education has increased in recent years, many curricula have not yet integrated Planetary Health modules into their study programmes (18). Therefore, we aim to develop an e-learning elective according to students‘ needs to implement knowledge and skills on Planetary Health and principles of transformative changes into health-related study programmes in Germany. Planetary Health being a rather new concept, there are only a few frameworks available to guide the conceptualization of Planetary Health education and more specifically electives at the academic level. However, our study results are in line with some concepts and topics given in the “Planetary Health education framework” of the Planetary Health Alliance (10). We chose an e-learning format for the elective as this format enables to reach students from various universities and fields of study. We believe that by implementing an e-learning format the elective will have the highest impact in driving broad awareness for and interest in this field. With this format, we will hopefully facilitate the implementation of Planetary Health education in curricula of various (health-related) fields of study at Bavarian and German universities in the near future. Furthermore, our findings can be a guidance for the development of further programmes in other disciplines and countries.

Limitations of this study include the unknown response rate, as the voluntary and snowball sampling strategy made it impossible to assert how many students actually received the invitation to participate in this study. In addition, the total population of Bavarian students in study programmes had been unknown as there is no overall documentation of the number and sociodemographic information of students in Bavarian study programmes available. Therefore, we are not able to assess a general tendency regarding the interest in participating in the survey based on gender, age or study programme. Furthermore, by also reaching participants beyond health-related fields of study, we had a diverse group of unevenly distributed study participants coming from a wide range of study programmes, making comparisons and general statements about small groups of studies, age or gender more difficult. Additionally, due to also accepting students into the survey who “self-identified” as relevant to this field of study, we cannot rule out a bias concerning the results of this survey. Lastly, we did not define the concept of Planetary Heath within our study, potentially creating difficulties in gaining an understanding of this concept while answering the survey questions.

We see further limitations in the design of the survey questionnaire. Due to a lack of detailed frameworks on which to base the compilation and choice of Planetary Health topics in this survey, we identified potential topics within the research team. Partly, the topics were derived from researchers' previous Planetary Health modules and partly by discussion in the research team. Since conducting this research, the German Alliance for Climate Change and Health has released a guideline for teaching options in Planetary Health (31). They propose 14 potential topics for general Planetary Health education to which our topics correspond well.

Strengths of this study are the high number of participants from a variety of health-related fields of study in Bavaria, Germany. Thereby we were able to gain new insights into which Planetary Health topics were most relevant for a broad audience of students. Additionally, various study programme offices contacted during the recruitment phase showed interest in the planned elective, increasing the chance that Planetary Health education will be added to curricula in Germany as a result of our study.

The survey suggests that there is ample room for integrating Planetary Health into health-related and science-based curricula, and with promising uptake by students. Given respondents' varied interests, curricular efforts should focus on relevance to students' field of study. Designing a Planetary Health online elective for a wide range of health-related fields of study programmes is an important step forward to strengthen inter- and transdisciplinary adoption. Additionally, the results of this study can be used as a framework and guidance for building Planetary Health courses and for setting thematic priorities according to students' fields of study and interest. The results from participants of other fields of study show that an even wider range of studies is relevant to Planetary Health and thus, its education should not only be limited to health-related fields of study. However, as this study focused on health-related fields of studies further research is necessary to focus specifically on other fields of study.

## Data availability statement

The raw data supporting the conclusions of this article will be made available by the authors, without undue reservation.

## Ethics statement

The studies involving human participants were reviewed and approved by Ethical Committee of Medical Faculty of LMU Munich. The patients/participants provided their written informed consent to participate in this study.

## Author contributions

MC, EH, CJ, VK, PS, and LS conceptualized the study. VK and PS performed the data collection and conducted the data analysis. MC, EH, CJ, IK-O, VK, PS, E-MS-S, and LS reviewed the data analysis and results. VK wrote the first draft of the manuscript. VK, PS, and MC have verified the underlying data. All authors had full access to all the data in the study and final responsibility for the decision to submit for publication, contributed to data interpretation, reviewed, and approved the manuscript.

## Funding

Virtuelle Hochschule Bayern (Virtual University Bavaria) (grant number: 21-I-05-11Reh1).

## Conflict of interest

The authors declare that the research was conducted in the absence of any commercial or financial relationships that could be construed as a potential conflict of interest.

## Publisher's note

All claims expressed in this article are solely those of the authors and do not necessarily represent those of their affiliated organizations, or those of the publisher, the editors and the reviewers. Any product that may be evaluated in this article, or claim that may be made by its manufacturer, is not guaranteed or endorsed by the publisher.
