# Supplementary material for: A cross-sectional study on the knowledge of and interest in Planetary Health in health-related study programmes in Germany
Source: Front Public Health. 2022 Oct 31;10:937854. doi: 10.3389/fpubh.2022.937854 (PMC9660317; doi:10.3389/fpubh.2022.937854)
Supplement: Supplementary file 1 [file Table_1.DOCX]

Supplementary Material

# Supplementary Data

## Supplementary Tables

| **University** | **Study programme** |
| --- | --- |
| Ansbach University of Applied Sciences | - Biomedical Engineering - Leadership (majoring in Health Science) |
| Augsburg University of Applied Sciences | - Business Psychology |
| Bundeswehr University Munich | - Psychology - Psychology majoring in Clinical Psychology and Psychotherapy - Sports Science: Health, Movement and Performance - Sport Sciences: Training, Health and Management in Sport |
| Catholic Foundation University of Applied Sciences (KSH) Munich | - Business Psychology - Education and Education Management in the Health System - Healthcare Management - Management of Social and Healthcare Institutions - Midwifery - Nursing - Nursing Education |
| Catholic University of Eichstaett-Ingolstadt | - Business and Psychology - Nursing Science - Psychology - Psychology with Specialization in School Psychology - Social Work |
| Coburg University of Applied Sciences and Arts | - Integrative Health Promotion - Midwifery, Health Promotion - Social Work |
| Deggendorf Institute of Technology | - Applied Psychology - Applied Sports Science - Applied Sports Science with Focus on Interprofessional Care in Sports - Global Public Health - Health Informatics - International Tourism Management / Health & Medical Tourism - Management in Health, Social and Rescue Services - Nursing - Organisational and Economic Psychology - Pedagogy in Rescue Services - Physician Assistant / Medical Care - Physiotherapy Dual Majoring in Kinesiology - Tourism Management - Vocational Pedagogy - Health & Care |
| DHGS University of Health and Sports Munich | - Medical Education - Medical Sports & Health Management - Sports Science |
| FOM University of Applied Sciences for Economics and Management Munich | - Business Psychology - Business Psychology & Consulting - Dentistry - Health Psychology & Medical Pedagogics - Medical Management - Nursing Management - Operational Health Management - Public Health - Social and Health Services Management |
| Friedrich-Alexander-University of Erlangen-Nuremberg | - Advanced Nursing Practice - Gerontology - Health and Medical Management - Health Business Administration - Health Management and Health Economics - Medical Process Management - Medicine - Molecular Medicine - Physical Activity and Health - Psychology - Science of Midwifery |
| Fresenius University of Applied Sciences Munich | - Business Psychology - Nutrition and Fitness in Prevention - Osteopathy - Physiotherapy - Psychology - Psychology & Digital Transformation |
| Hof University of Applied Sciences | - Education for Health Professions |
| HSD Doepfer - University of Applied Sciences Regensburg | - Applied Therapy Science - Health Education Studies - Medical Education - Physician Assistance - Psychiatric Care |
| IB School of Health and Social Affairs Munich | - Applied Psychology |
| IU International University Augsburg | - Applied Health Services Research - Business Psychology - Childhood Education - Curative and Inclusion Education - Dietetics - Gerontology - Health Care and Nursing Education - Health Economics - Health Education Studies - Health Management - Health Psychology - Nursing - Nursing Education - Nursing Management - Nutrition Science - Physiotherapy - Public Health - Social Work |
| Kempten University of Applied Sciences | - Biomedical Engineering - Gerontological Nursing and Therapy - Health Services Management - Healthcare Informatics - Social and Health Services Management - Social Economics - Social Work (majoring in Health Promotion and Disease Prevention) |
| Landshut University of Applied Sciences | - Dentistry - Midwifery - Physician Assistant |
| LMU Munich | - Epidemiology - International Health - Medicine - Neuro-Cognitive Psychology - Pharmaceutical Sciences - Psychology - Public Health - School Psychology - Veterinary Medicine |
| Lutheran University of Applied Sciences Nuremberg | - Child Development, Education, Health - Economics in Social and Health Services - Health- and Care Education - Health and Care Management - Nursing - Psychology - Social Work |
| Macromedia University Munich | - Business Psychology |
| New-Ulm University of Applied Sciences | - Business Studies in Healthcare Management - Digital Healthcare Management - Leadership and Management in Healthcare - Management for Health and Nursing Professions - Physician Assistant |
| Nuremberg Institute of Technology | - Applied Organizational Psychology - Science of Midwifery - Social Work |
| OTH Regensburg – Technical University of Applied Sciences | - Advanced Nursing Practice - Applied Psychology - Biomedical Neuroscience - Care Management - Corporate Management for Health Professions - Digital Health Management - Health Care Sciences - Healthcare Management - Medical Law - Medical Technology - Nursing - Physician Assistance |
| Technical University of Munich | - Computational Life Sciences - Ergonomics – Human Factors Engineering - Food Chemistry - Health Science - Health Science - Prevention and Health Promotion - Life Sciences Nutrition - Nutrition and Biomedicine - Sport and Exercise Science - Traditional Chinese Medicine - Vocational Education Nutrition and Home Economics (Teaching at Vocational Schools) |
| THI - University of Applied Sciences Ingolstadt | - Health Economics - Health Management - Information Management in Healthcare - Life Science Management - Management in Health Professions - Media Psychology and Digital Business |
| University of Applied Management Munich | - Digital Psychology - Healthcare Management - Media Psychology - Psychology in Sport and Performance |
| Ulm University of Applied Sciences | - Business Psychology |
| University of Applied Sciences Munich | - Applied Health- and Therapy Sciences - Applied Nursing Science - Mental Health - Social Changes and Participation - Social Work - Social Work - Diagnostics, Counselling and Intervention |
| University of Applied Sciences of the Middle Class (FHM) Bamberg | - Business Psychology - Medical Technology & Management - Physician Assistance - Psychology |
| University of Applied Sciences Wuerzburg-Schweinfurt | - Social Work |
| University of Augsburg | - Medical Information Science - Medicine - Psychology |
| University of Bamberg | - Food & Health Sciences - Psychology with Specialization in School Psychology |
| University of Bayreuth | - Dentistry - Food Quality and Safety - Global Food, Nutrition and Health - Health Care Management - Health Economics |
| University of Regensburg | - Biomedicine - Medicine - Pharmacy - Psychology - Special Needs Education |
| University of Wuerzburg | - Dentistry - Medicine - Pharmacy - Psychology - Translational Medicine - Translational Neuroscience |
| Weihenstephan-Triesdorf University of Applied Sciences | - Animal Health Management - Nursing - Nutrition and Food Supply Management - Organic Food Products & Business |
| Wilhelm Loehe University of Applied Sciences Fuerth | - Psychology - Social and Health Services Management - Social Work - Vocational Education in Health - majoring Nursing, Rescue Services/Aesthetic Assistant - Vocational Education in Health Care |

**Supplementary Table 1.** Description of contacted universities and degree programmes.

| **Sociodemographic Data** | |
| --- | --- |
| Age | a. 18 – 22 years  b. 23 – 27 years  c. 28 – 32 years  d. 33 – 37 years  e. 38 – 42 years  f. 43 years and above |
| Gender | a. Female  b. Male  c. Divers  d. No answer |
| In which study programme are you currently enrolled? | a. Medicine  b. Public health  c. Epidemiology  d. Health sciences  e. Nursing sciences  f. Psychology  g. Physiotherapy  h. Others (*free text*) |
| What degree are you pursuing with the study programme you are currently enrolled in? | a. Bachelor  b. Master  c. PHD  d. State examination  e. Others (*free text*) |
| **Planetary Health in General** | |
| Have you heard of the discipline Planetary Health? | a. Yes  b. No  c. No answer |
| *If yes*: What does Planetary Health mean? | Free text |
| Are you interested in learning more about Planetary Health? | a. Yes  b. More likely  c. Less likely  d. No  e. No answer |
| *If yes:* How have you been in touch with Planetary Health topics so far? | a. Self-interest  b. Social media  c. Print media  d. (University) education  e. Extracurricular/ non-university training  f. Friends/ acquaintances  g. Other (*free text*) |
| Which topics (in the area of Planetary Health) are you most interested in? Please select up to six answers. | a. General Associations between Health and Climate/Environment  b. Climate Change  c. Planetary Boundaries  d. Extreme Weather Events and Health (e.g. heat waves)  e. Climate-induced Transmission of Infectious Diseases (e.g. diarrheal diseases in warm summers due to Salmonella, Campylobacter)  f. Climate-induced Transmission of Vector-borne Diseases (e.g., malaria or dengue virus caused by mosquitoes)  g. Climate Change and Non-communicable Diseases (e.g. allergies)  h. Mental Health  i. (Micro)plastic and Health  j. Biodiversity Crisis  k. Forest Resources and Health  l. Planetary Health Diet  m. Health-related Influences of the Atmospheric Environment  n. Energy Systems  o. Climate-friendly Health Sector  p. Mobility for People and Planet  q. Climate-friendly Urban Development  r. Ethical Aspects  s. The Global South  t. Initiating Transformational Processes  u. Health Literacy/Sustainability Competence  v. Systems Research (e.g. Concept of Tipping Points)  w. Other topics (*free text*) |
| **Planetary Health in Education** | |
| How important is the implementation of Planetary Health topics in your studies to you? | a. Very important  b. Rather important  c. Rather unimportant  d. Unimportant  e. No answer |
| Would you choose a Planetary Health online course, if it were available as an elective at your university? | a. Yes  b. More likely  c. Less likely  d. No  e. No answer |

**Supplementary Table 2.** Study questionnaire.
